# Green Synthesis of Gold and Silver Nanoparticles Using Leaf Extract of *Capsicum chinense* Plant

**DOI:** 10.3390/molecules27051692

**Published:** 2022-03-04

**Authors:** Diego Alberto Lomelí-Rosales, Adalberto Zamudio-Ojeda, Oscar Kevin Reyes-Maldonado, Morelia Eunice López-Reyes, Georgina Cristina Basulto-Padilla, Edgar José Lopez-Naranjo, Víctor Manuel Zuñiga-Mayo, Gilberto Velázquez-Juárez

**Affiliations:** 1 Departamento de Química, Centro Universitario de Ciencias Exactas e Ingenierías, Universidad de Guadalajara, Blvd. Marcelino García Barragán #1421, Guadalajara 44430, Jalisco, Mexico; diego.lomeli4077@academicos.udg.mx (D.A.L.-R.); oscar.reyes@alumnos.udg.mx (O.K.R.-M.); morelia.lopez@academicos.udg.mx (M.E.L.-R.); georgina.basulto@alumnos.udg.mx (G.C.B.-P.); 2 Departamento de Física, Centro Universitario de Ciencias Exactas e Ingenierías, Universidad de Guadalajara, Blvd. Marcelino García Barragán #1421, Guadalajara 44430, Jalisco, Mexico; adalberto.zojeda@academicos.udg.mx; 3 Departamento de Ingeniería de Proyectos, Universidad de Guadalajara, José Guadalupe Zuno # 48, Zapopan 45101, Jalisco, Mexico; edgar.lopezn@academicos.udg.mx; 4 CONACyT-Instituto de Fitosanidad, Colegio de Postgraduados, Campus Montecillo, Texcoco 56230, Estado de México, Mexico

**Keywords:** metal nanoparticles, antioxidant, antimicrobial, chilli, stabilizer

## Abstract

So far, several studies have focused on the synthesis of metallic nanoparticles making use of extracts from the fruit of the plants from the genus Capsicum. However, as the fruit is the edible, and highly commercial, part of the plant, in this work we focused on the leaves, a part of the plant that is considered agro-industrial waste. The biological synthesis of gold (AuNPs) and silver (AgNPs) nanoparticles using aqueous extracts of root, stem and leaf of *Capsicum chinense* was evaluated, obtaining the best results with the leaf extract. Gold and silver nanoparticles synthesized using leaf extract (AuNPs-leaf and AgNPs-leaf, respectively) were characterized by UV-visible spectrophotometry (UV-Vis), Fourier Transform Infrared Spectroscopy with Attenuated Total Reflection (FTIR-ATR), X-ray Photoelectron Spectroscopy (XPS), Ultra Hight Resolution Scanning Electron Microscopy coupled to Energy-Dispersive X-ray spectroscopy (UHR-SEM-EDX) and Transmission Electron Microscopy (TEM), and tested for their antioxidant and antimicrobial activities. In addition, different metabolites involved in the synthesis of nanoparticles were analyzed. We found that by the use of extracts derived from the leaf, we could generate stable and easy to synthesize AuNPs and AgNPs. The AuNPs-leaf were synthesized using microwave radiation, while the AgNPs-leaf were synthesized using UV light radiation. The antioxidant activity of the extract, determined by ABTS, showed a decrease of 44.7% and 60.7% after the synthesis of the AuNPs-leaf and AgNPs-leaf, respectively. After the AgNPs-leaf synthesis, the concentration of polyphenols, reducing sugars and amino acids decreased by 15.4%, 38.7% and 46.8% in the leaf extract, respectively, while after the AuNPs-leaf synthesis only reducing sugars decreased by 67.7%. These results suggest that these groups of molecules are implicated in the reduction/stabilization of the nanoparticles. Although the contribution of these compounds in the synthesis of the AuNPs-leaf and the AgNPs-leaf was different. Finally, the AgNPs-leaf inhibited the growth of *S. aureus*, *E. coli*, *S. marcescens* and *E. faecalis.* All of them are bacterial strains of clinical importance due to their fast antibiotic resistance development.

## 1. Introduction

Nanotechnology is one of the fastest-growing fields in recent years due to its potential applications. Particularly, metallic nanoparticles, due to their physicochemical properties as they have different applications as vehicles for the delivery of drugs and nucleic acids, biomolecules, antimicrobials and their potential in cancer diagnosis and therapy, among others [1,2,3]. The methodologies for the synthesis of nanoparticles are classified as: physical, chemical, and biological. Physical methodologies consume a large amount of energy-raising production costs, while chemical methodologies make use of organic solvents and toxic reagents limiting their application in medicine and producing harmful waste to the environment [3,4]. In contrast, biological methods or green synthesis represent an alternative that is environmentally friendly, inexpensive, easy to use, with little to low harmful potential and compatibility with living organisms [2,5]. Biological methods are characterized by the use of organisms (or products derived from them) for the synthesis of nanoparticles. Microorganisms such as bacteria, fungi and algae, as well as extracts from these, are regularly used [4].

So far, a wide range of vegetal species from different families have been used for the synthesis of nanoparticles [5,6,7]; between them, different species from the genus Capsicum [7,8]. The genus Capsicum belongs to the Solanaceae family, and it counts with [9,10,11,12] species among which five have been domesticated and harvested. *Capsicum annuum*, *C. frutescens*, *C. pubescens*, *C. baccatum* and *C. chinense* are species native to the tropical and template zones of America, including Mexico. The fruit of these species has many names, “chilli pepper” being the most common [13,14,15].

Chilli is a crop of great economic importance, and in 2019 around 42 million tons were produced that were marketed as fresh or dried chilli, ranking seventh worldwide for harvested vegetables (FAO, 2019). This vegetable is widely used in traditional cuisine providing flavor, aroma and color to various dishes around the world, and in addition, it is used in the food and cosmetics industry. It also has potential applications in medicine because it contains compounds such as flavonoids, vitamins C and E, carotenoids, minerals and especially capsaicinoids with nutraceutical, analgesic, antimicrobial, anti-obesity and anticarcinogenic effects [16,17,18].

Extracts obtained from different species of the genus Capsicum (*C. annuum*, *C. frutescens*, *C. baccatum* and *C. chinense*) were shown to be a good option for the green synthesis of nanoparticles [15,19,20,21,22,23,24,25]. However, few studies have used extracts from a plant organ other than the fruit, such as leaves [26,27]. This represents a problem as the fruit is the only part of the plant commonly traded, while the rest of the plant is considered agricultural waste after harvest. On the other hand, it is well known that the reduction process of nanoparticles by green synthesis, using phytochemical extracts, is mediated by the reducing capacity of several compounds [28,29]. Recently, several authors have evaluated the antioxidant capacity of the nanoparticles once synthesized [10,11,12]. However, there are very few works where authors try to evaluate the changes in the antioxidant activity after the synthesis of nanoparticles or the groups of compounds implicated in the reduction process. For this reason, we decided to explore the potential of the extracts obtained from various parts of the *C. chinense* plant (root, stem, and leaf) that do not have commercial use in order to find the conditions that would allow for the use of extracts derived from these wastes as reducing or stabilizing agents in the green synthesis of gold and silver nanoparticles. Likewise, we monitored the chemical composition of the extracts at the beginning and the end of the green synthesis carried out under optimal conditions for the generation of nanoparticles. Additionally, the antimicrobial activity against *S. aureus*, *E. coli*, *S. marcescens* and *E. faecalis* was evaluated.

## 2. Results

### 2.1. Synthesis and Stability of the AuNPs-Leaf

Plants of *C. chinense* were collected in the fruit setting stage, and the samples were separated into three groups: root, stem, and leaf. The aqueous extracts (root-extract, stem-extract and leaf-extract) obtained from these three samples presented coloration from colorless to light yellow, indicating the presence of biomolecules from the plant.

Using the extracts previously prepared, an experiment was carried out where 1 mL of each extract was mixed with three different amounts (8.05 × 10^−3^, 1.60 × 10^−2^ and 2.4 × 10^−2^ mmol) of tetrachloroauric acid (HAuCl_4_) and several exposure times to UV radiation (5–300 min) were tested. In this experiment, we did not obtain any favorable results.

Then we decided to explore the effect of microwave radiation for synthesizing AuNPs. Again, the three extracts were evaluated (1 mL of each) with the same amounts of HAuCl_4_ used before but with an exposure time of 15 s to microwave radiation (Figure S1). Synthesis with the three extracts at 8.05 × 10^−3^ mmol of HAuCl_4_ showed favorable results for the generation of AuNPs.

**Figure 1:**
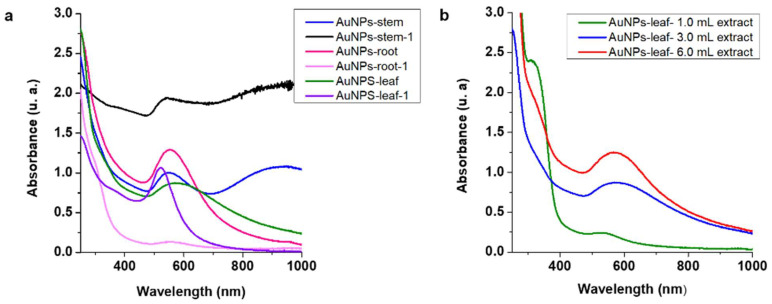
UV-vis absorption spectra of AuNPs. (**a**) AuNPs obtained from different extracts (stem, root and leaf) were analyzed at time zero (AuNPs-component) and one hour after synthesis (AuNPs-component-1) (**b**) Absorption spectra of AuNPs-leaf extract with different volumes of leaf extract.

Figure 1a shows the UV-vis absorption spectra of the AuNPs prepared from the leaf (AuNPs-leaf), the stem (AuNPs-stem) and the root (AuNPs-root) at time zero (immediately after being synthesized) and one hour after synthesis (AuNPs-leaf-1, AuNPs-stem-1, and AuNPs-root-1). According to the absorption spectrum measured at time zero, the AuNPs-root presented a single absorption curve at 552 nm, possibly corresponding to spherical nanoparticles. However, the absorption curve at 552 nm diminished in less than one hour, indicating a loss of nanoparticles (AuNPs-root-1).

For the AuNPs-stem sample, two absorption curves were observed at 548 and 930 nm, suggesting the presence of nanoparticles with non-spherical morphologies. However, one hour after synthesis an anomalous absorption curve was observed (AuNPs-root-1), and “gold buttons” appeared at the bottom of the reaction vial (Figure 1a), indicating an uncontrolled growth of nanoparticles.

Meanwhile, the AuNPs-leaf presented an absorption curve at 570 nm at time zero, nevertheless, 1 h later, a decrease in the maximum absorption curve (523 nm) and a better symmetry were observed (Figure 1a). This result was an indication that the formation of AuNPs had not been completed. For these reasons, we decided to discard the AuNPs-stem and AuNPs-root synthesis routes and work exclusively with the synthesis of AuNPs-leaf.

Finally, a third experiment was carried out to find the best volume of extract for the optimal synthesis of the AuNPs-leaf. The amount of HAuCl_4_ (8.05 × 10^−3^) and the exposure time were kept fixed and only the volume of leaf extract was varied this time (1 mL, 3 mL 6 mL).

The absorption curves for the AuNPs-leaf obtained with different volumes of extract (1.0 mL, 3.0 mL, and 6.0 mL) are shown in Figure 1b. The results showed that 6.0 mL of extract was the optimal volume for the synthesis of the AuNPs-leaf. It should be noted that if an excess of extract (>6.0 mL) was used, optimal stabilization of the nanoparticles was not achieved (data not shown).

**Figure 2:**
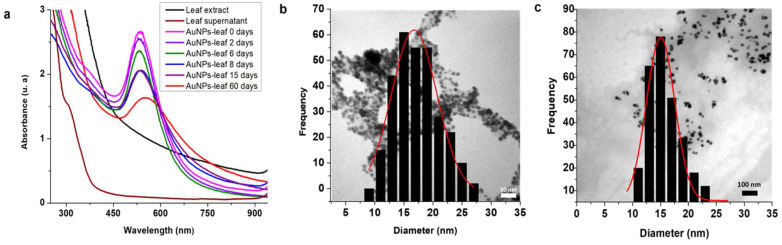
Stability and size of AuNPs-leaf. (**a**) Stability of AuNPs-leaf at 0, 2, 6, 8, 15 and 60 days after synthesis. It was not possible to appreciate any absorption for neither the leaf extract nor the supernatant (after AuNPs purification) that could interfere with the absorbances by AuNPs-leaf (**b**) Particle size distribution from TEM imaging of the AuNPs-leaf at zero days after synthesis, where we can observe spherical nanoparticles of 16.76 ± 0.32 nm. (**c**) Particle size distribution from TEM imaging of the AuNPs-leaf at 15 days after synthesis, where we can observe spherical nanoparticles of 15.05 ± 0.27 nm.

The stability of the AuNPs-leaf was monitored by UV-vis spectroscopy and transmission electron microscopy (TEM), (Figure 2). The AuNPs-leaf were stable for up to 15 days, as evidenced by the variations in the absorption curve at 552 nm (Figure 2a). To corroborate the UV-vis observations, the AuNPs were characterized by transmission electron microscopy (TEM) on the same day of their synthesis, where particle sizes of 16.76 ± 0.32 nm were observed (Figure 2b); and 15 days after their preparation, they presented similar sizes 15.05 ± 0.27 nm (Figure 2c).

### 2.2. Synthesis and Stability of AgNPs-Leaf

Once the conditions for the synthesis of AuNPs-leaf were established. We proceeded to prepare the AgNPs-leaf. Initially, the effect of microwave radiation was evaluated, where the leaf extract volume (1 mL) and the amount (8.05 × 10^−3^ mmol) of silver nitrate (AgNO_3_) were kept constant. We could not observe any change in the coloration of the solution and thus no nanoparticle formation. Then, we tested the effect of the UV light radiation (Figure S2), finding that at 5 min of exposition and 8.05 × 10^−^^3^ mmol of AgNO_3_, a brown darkening in the solution was observed, indicating the formation of the AgNPs. Based on these results, in the following experiments, UV light radiation and 8.0 × 10^−^^3^ mmol of AgNO_3_ were used to synthesize AgNPs.

Once the UV light was selected as a source of radiation, an experiment for determining the optimal radiation exposure time was performed. For this purpose, several samples (1 mL of leaf extract and 8.0 × 10^−^^3^ mmol of AgNO_3_) were exposed to UV light radiation for 5 to 180 min. Every 5 min, one sample was removed from the UV light radiation and stored for further UV-vis spectroscopy analysis to determine which sample had the highest concentration of nanoparticles (evidenced by a higher absorbance). Results indicated that 20 min of UV light radiation was optimal for the AgNPs-leaf synthesis. A prolonged exposure period (3 h) resulted in nanoparticles destabilization, presenting decomposition of the formed nanoparticles (macro-metric silver precipitation and a greenish-gray coloration in solution) (data not shown).

**Figure 3:**
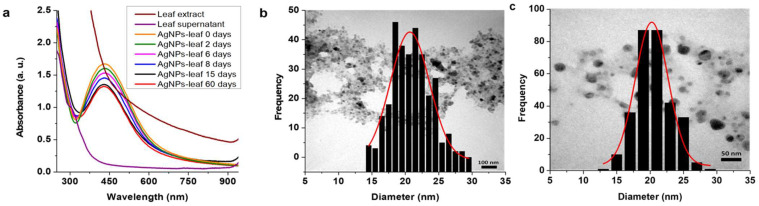
Stability and size of AgNPs-leaf. (**a**) Stability of AgNPs-leaf at 0, 2, 6, 8, 15 and 60 days after synthesis. It was not possible to appreciate any absorption for neither the leaf extract nor the supernatant (after AgNPs purification) that could interfere with the absorbances by AgNPs-leaf (**b**) Particle size distribution from TEM imaging of the AgNPs-leaf at zero days after synthesis, where we can observe spherical nanoparticles of 20.67 ± 0.26 nm. (**c**) Particle size distribution from TEM imaging of the AgNPs-leaf at 15 days after synthesis, where we can observe spherical nanoparticles of 20.24 ± 0.24 nm.

Like the AuNPs-leaf, the AgNPs-leaf were purified in the same way, and a stability study was performed. Figure 3a shows the UV-vis absorption curve of the AgNPs-leaf where we focused on analyzing the variations in the wavelength of maximum absorption at 430 nm. The maximum wavelength absorption curve at 15 and 60 days were very similar, indicating a better stabilization for the AgNPs-leaf than for the AuNPs-leaf.

Additionally, the AgNPs-leaf were characterized by TEM on the same day of their synthesis, where particle sizes of 20.67 ± 0.26 nm were observed (Figure 3b); and 15 days after their preparation, presenting similar sizes, 20.24 ± 0.24 nm (Figure 3c).

### 2.3. SEM-EDX Analysis of the AuNPs-Leaf and the AgNPs-Leaf

**Figure 4:**
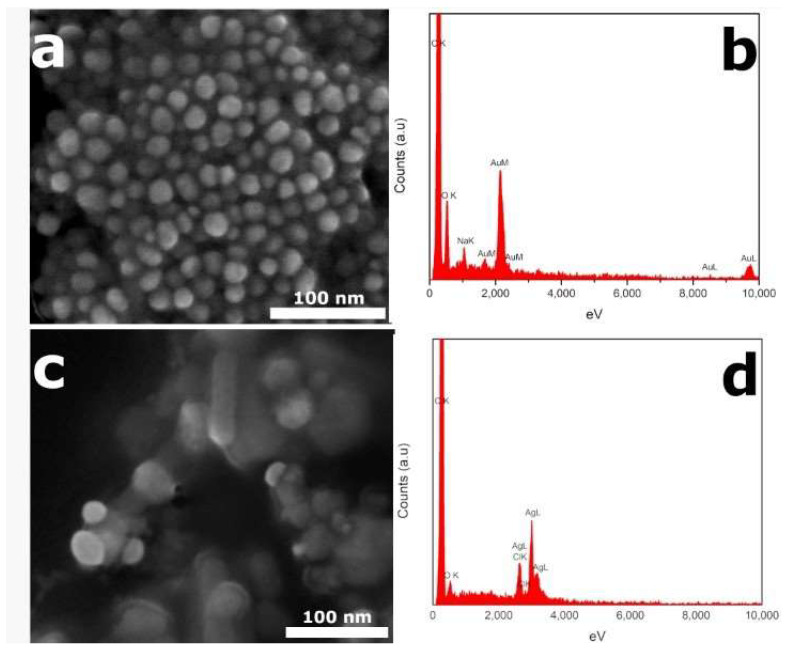
SEM-EDX analysis of AuNPs-leaf and AgNPs-leaf. (**a**) SEM image of AuNPs-leaf sample representing the particle distribution and morphology at 400 kX magnification. (**b**) EDX spectrum of the AuNPs-leaf sample in which the L and M signal lines from the gold atoms, can be observed. (**c**) SEM image of AgNPs-leaf sample representing the particle distribution and morphology at 350 kX magnification. (**d**) EDX spectrum of the AuNPs-leaf sample in which the L and M signal lines from the gold atoms, can be observed. White bars in the figures indicate the scale of 100 nm.

In addition to TEM microscopy, which allowed us to determine the average size distribution of nanoparticles, we performed an analysis by Ultra-High Resolution Scanning Electron Microscopy coupled to Energy-Dispersive X-ray spectroscopy (UHR-SEM-EDX). This technique allowed us to determine the chemical composition of the synthesized nanoparticles. Through SEM-EDX, we observed homogeneous size distribution of the AuNPs-leaf and quasi-spherical morphologies (Figure 4a). These data are in good agreement with TEM observations.

Furthermore, according to the EDX spectra shown in Figure 4b, it could be determined that the AuNPs-leaf are formed by gold atoms, with some traces of sodium, which could have come from the leaf extract. While for the AgNPs-leaf, we could observe some nanoparticles with elongated morphologies; however, most of the nanoparticles showed almost spherical symmetries (Figure 4c). Additionally, with the EDX technique, we observed the L-line signal of the silver atoms that make up the nanoparticles (Figure 4d).

### 2.4. Antioxidant Activity of Leaf-Extract and Nanoparticle Supernatants

Antioxidant activity was determined by three methodologies, ABTS, DPPH, and FRAP. A reference curve with Trolox was used, and the results were expressed in units of Trolox equivalents (TE). The change in antioxidant activity of the extract and supernatants (obtained after synthesis) was monitored.

**Figure 5:**
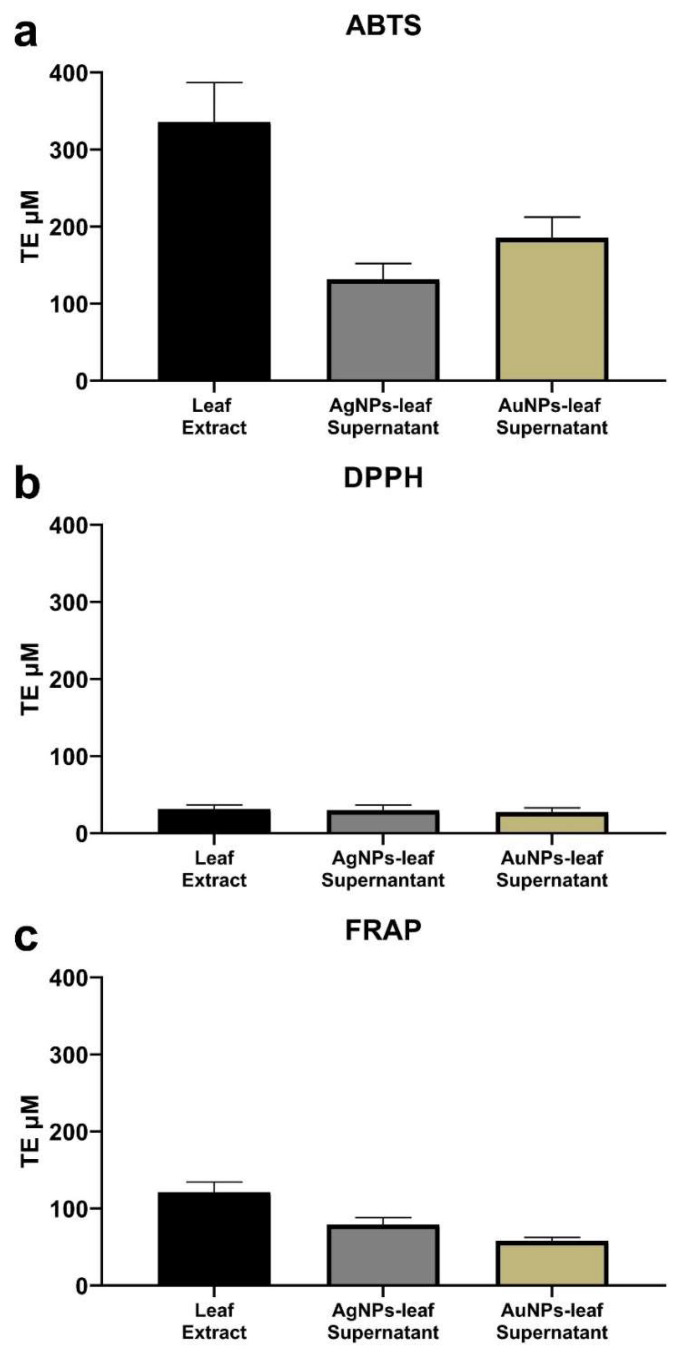
Antioxidant activity of leaf extract, silver (AgNPs-leaf) and gold (AuNPs-leaf) supernatants by three methodologies. (**a**) Antioxidant activity of AgNPs-leaf and AuNPs-leaf measured by ABTS. (**b**) Antioxidant activity of AgNPs-leaf and AuNPs-leaf measured by DPPH. (**c**) Antioxidant activity of AgNPs-leaf and AuNPs-leaf measured by FRAP. A standard Trolox curve in a range of 0–400 µM was used for all measurements. The results were expressed as μmol Trolox equivalent (TE µM). Values are means ± standard deviation (*n* = 3). Statistical analysis was performed by a one-way ANOVA, with Tukey’s multiple comparisons post-test. Data within the same chart sharing different letters are significantly different at *p* < 0.05.

The value of the initial antioxidant activity for the leaf extracts used for the synthesis was: 335.8 ± 51.3 µM TE, 31.4 ± 5.4 µM TE and 120.9 ± 12.6 µM TE measured by ABTS, DPPH and FRAT methods, respectively (Figure 5a–c). On the other hand, when the antioxidant activity in the AgNPs-leaf and AuNPs-leaf supernatants were analyzed by the ABTS method, a significant change was observed with antioxidant activity values of 131.8 ± 20.3 µM TE for the AgNPs-leaf supernatant and 185.5 ± 26.9 µM TE for the AuNPs-leaf supernatant, which represent a decrease of 60.7% and 44.7% of the original activity value (extract), respectively (Figure 5a). Moreover, the results quantified with the FRAP methodology also show a significant decrease in the antioxidant activities of the AgNPs-leaf (79.1 ± 9.1 µM TE) and AuNPs-leaf (57.9 ± 4.7 µM TE) supernatants that represent a reduction of the initial antioxidant activity (extract) of 34.5% and 52.1%, respectively (Figure 5c). There were no significant differences at 95% confidence evaluated with ANOVA for the samples analyzed by DPPH (Figure 5b). Together these data indicate that those molecules detected by the ABTS and FRAP methodology are involved in reducing the AgNPs-leaf and AuNPs-leaf, but those molecules sensed by DPPH are not.

### 2.5. Phytochemical Screening of the Extracts before and after Nanoparticles Synthesis

The leaf extract and the supernatants were evaluated for various metabolites, such as proteins, flavonoids, amino acids, polyphenols and reducing sugars. The phytochemical analysis of the extract was performed before and after the synthesis of nanoparticles. Before synthesis, the extract was analyzed directly. After synthesis, the aqueous phase of the sample was recovered by centrifugation (supernatant) and then analyzed.

**Figure 6:**
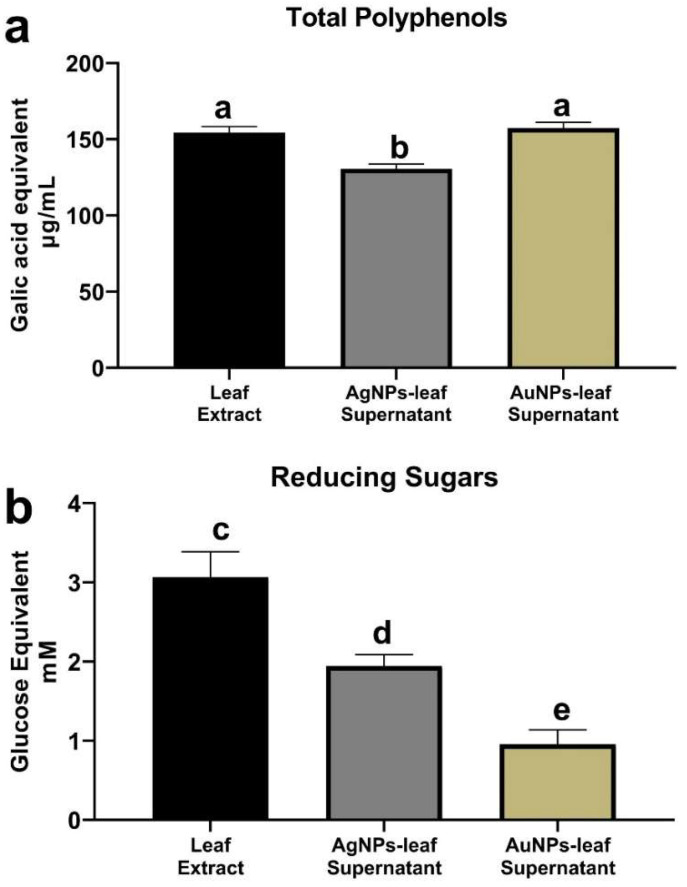

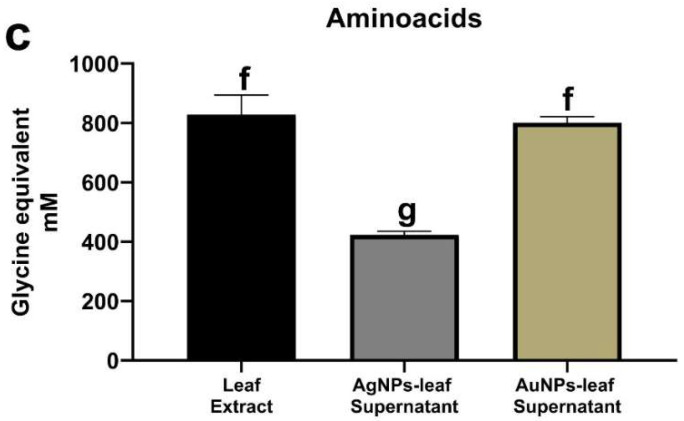
Phytochemical screening (Total polyphenols, reducing sugars, amino acids) of the leaf extracts and the silver (AgNPs-leaf) and gold (AuNPs-leaf) supernatants. (**a**) Concentration of total polyphenols estimated by the Folin–Ciocalteau method. (**b**) Concentration of the reducing sugars estimated by the Miller method. (**c**) Concentration of total amino acids estimated by the ninhydrin method. Values are means ± standard deviation (*n* = 3). Statistical analysis was performed by a one-way ANOVA, with Tukey’s multiple comparisons post-test. Data within the same chart sharing different letters are significantly different at *p* < 0.05.

We were unable to detect either protein or flavonoids in the samples by the Bradford and aluminum trichloride (AlCl_3_) methods, respectively. Total polyphenols were determined by the Folin–Ciocalteau method using gallic acid equivalent (GAE) as reference. The leaf extract had a value of 154.4 ± 4.0 µg GAE/mL, while values of 130.6 ± 5.1 µg GAE/mL and 157.3 ± 3.7 µg GAE/mL were obtained from the AgNPs-leaf and the AuNPs-leaf supernatants, respectively (Figure 6a). For the AgNPs-leaf supernatant, this represents a decrease of 15.4% in polyphenol concentration compared to the leaf extract. The AuNPs-leaf supernatant did not show a statistically significant change.

The Miller method was used to estimate the amount of reducing sugars with glucose equivalent (GluE) as reference. The leaf extract contained 3.1 ± 0.3 mM GluE. For AgNPs-leaf supernatant, a concentration of 1.9 ± 0.1 mM GluE was estimated, while the AuNPs-leaf supernatant value was 1.0 ± 0.2 mM GluE, indicating a decrease of 38.70% and 67.74% compared to the control, respectively (Figure 6b).

Finally, free amino acids were estimated by the ninhydrin method using glycine equivalent (GlyE) as reference. The estimated concentration of free amino acids was 794.3 ± 174.7 mM GlyE for the leaf extract, 422.5 ± 25.4 mM GlyE for the AgNPs-leaf supernatant, and 800.9 ± 41.5 mM GlyE for the AuNPs-leaf supernatant (Figure 6c), indicating a decrease of 46.80% in the AgNPs-leaf supernatant compared to the leaf extract. In contrast, in the AuNPs-leaf supernatant, there was no statistically significant change.

### 2.6. Characterization by FTIR-ATR and XPS of the AgNPs-Leaf and AuNPs-Leaf

**Figure 7:**
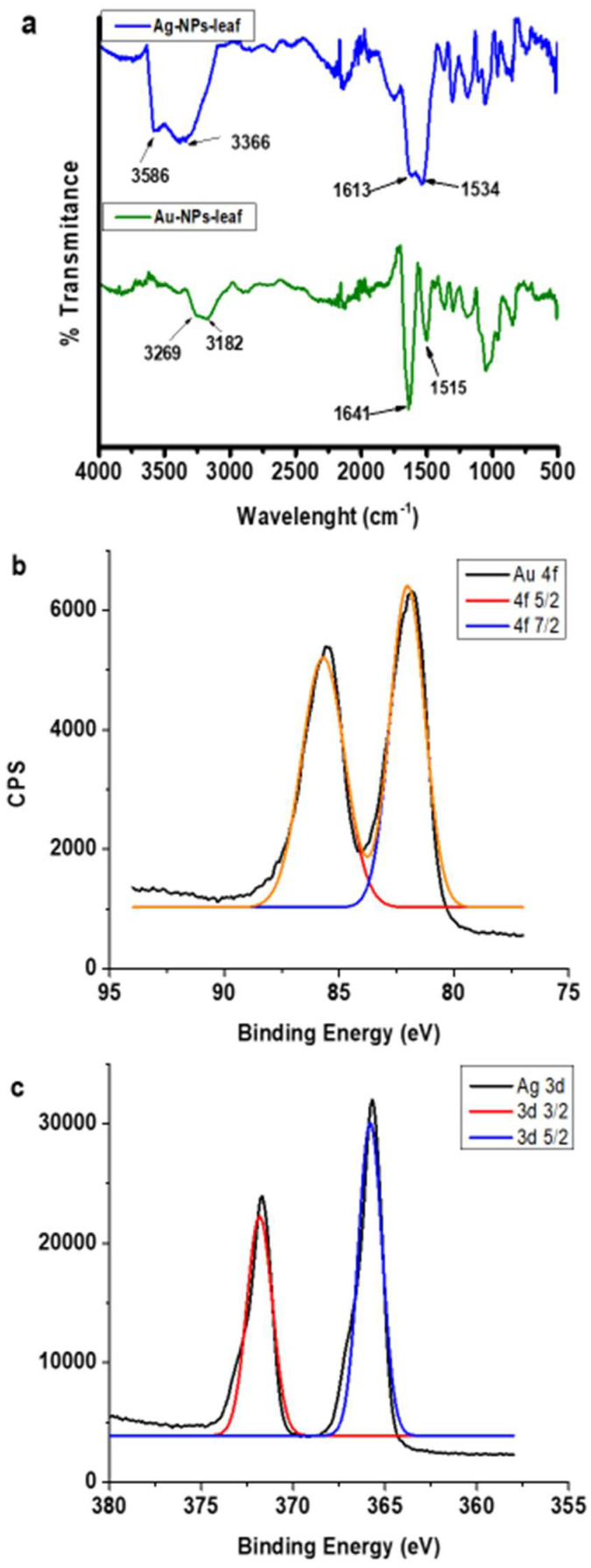
Spectroscopic analysis of AuNPs-leaf and AgNPs-leaf. (**a**) FTIR-ATR spectra of AgNPs-leaf and AuNPs-leaf. Arrows point to the associated bands of NH symmetric bending (1534 cm^−1^) and the NH antisymmetric bending (1613 cm^−1^) for AgNPs-leaf. Similar signals were found for the AuNPs-leaf, but with slight shifts to 1515 cm^−1^ and 1641 cm^−1^ respectively. (**b**) Au 4f XPS spectra of AuNPs-leaf. (**c**) Ag 3d XPS spectra of AgNPs-leaf.

The metal nanoparticles (MNPs) obtained in this work were quite stable due to the excellent coordination of the components of the extract itself. They were able to dry without presenting decomposition to be analyzed by FTIR-ATR spectroscopy and X-ray Photoelectron Spectroscopy (XPS). The spectra are shown in Figure 7.

When interpreting the FTIR-ATR spectrum for the AgNPs-leaf, bands are observed at 3596 cm^−1^ corresponding to the N–H and O–H stretch regions from 3300 to 3600 cm^−1^. A pair of characteristic signals were found, identified as the symmetric bending at 1534 cm^−1^ (NH_3_) and antisymmetric bending at 1613 cm^−1^ (NH_3_, shoulder). These bands correspond to the vibrational modes of the amino groups, according to previously reported data [30]. The band at 1613 cm^−1^ may also be due to the contribution of the anti-symmetric stretching mode of the carboxylate group (COO^−^) (Figure 7a).

The presence of the COO^−^ and NH groups suggests that the MNPs are overlayed by amino acids or a mixture of amino acids and oxidized sugars. Similar signals were found for the AuNPs-leaf, but with slight shifts (possibly due to the coordination of the stabilizer to the metal surface in the nanoparticle).

In order to corroborate the presence of amino acids overlaying the MNPs, purified nanoparticles were analyzed by the ninhydrin method. The results showed that the AgNPs-leaf contain 129.3 ± 14.3 mM GlyE, while the AuNPs-leaf contain 89.6 ± 7.0 mM GlyE. These data indicated that the AgNPs-leaf and the AuNPs-leaf retain 16.2% and 11.2% of the free amino acids contained in the original leaf extract, respectively.

The chemical composition and the oxidation state (Au^0^ and Ag^0^) of the surface of the obtained AuNPs-leaf and the AgNPS-leaf were analyzed by XPS (Figure 7b,c). The deconvolution of full XPS spectra confirmed the presence of nitrogen, carbon, and oxygen atoms, at the surface of the nanostructures. All the data together suggest the possible presence of amino acids, and also the absence of sulfur amino acids, since the presence of sulfur atoms was not observed in any of the analyses.

### 2.7. Antimicrobial Activity of the NPs-Leaf

In order to evaluate the possible applications of the MNPs obtained in this work, we tested its antimicrobial activity against *Staphylococcus aureus*, *Enterococcus faecalis*, (Gram-positive bacteria) *Escherichia coli* and *Serratia marcescens* (Gram-negative bacteria). Nanoparticles obtained by biological (MNPs-leaf) and chemical (MNPs-chem) synthesis were used in this assay to determine if the biological synthesis had any impact on the antimicrobial activity of the nanoparticles. The inhibition zones generated by the AgNPs-leaf and the AgNPs-chem against *S. aureus*, *E. faecalis*, *E. coli* and *S. marcescens* can be seen in Table 1.

**Figure 8:**
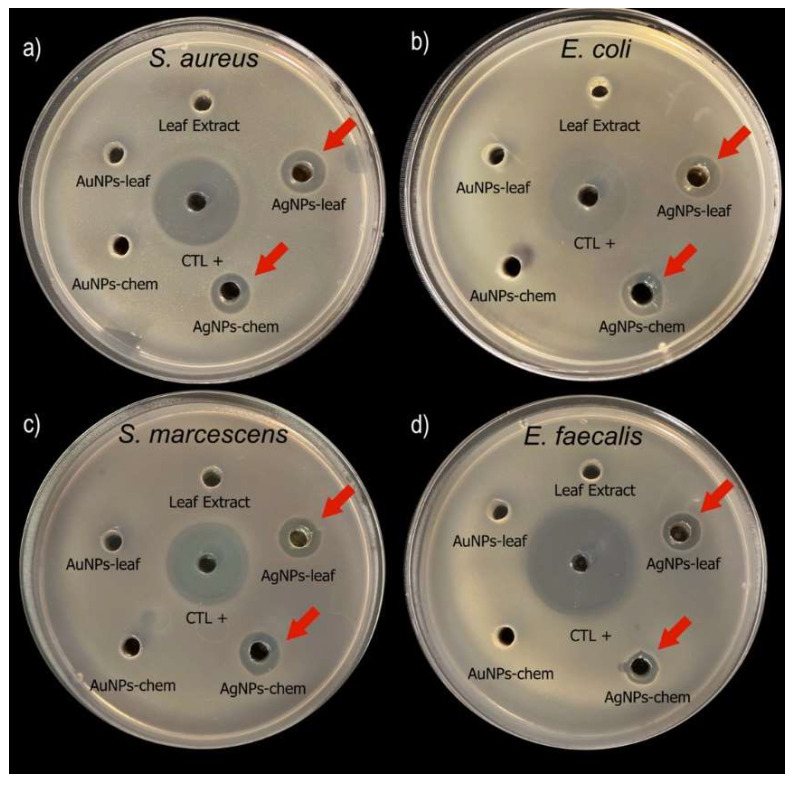
Antimicrobial activity of the nanoparticles obtained by biological and chemical synthesis. The AgNPs-leaf and AgNPs-chem showed antimicrobial activity against *S. aureus* (**a**), *E. coli* (**b**), *S. marcescens* (**c**) and *E. faecalis* (**d**). The AuNPs-leaf and AuNPs-chem did not show antimicrobial activity against any strain. All the MNPs used in this study contained the same amount of metal in solution to make a fair comparison of activity. Kanamycin was used as a positive control (CTL+).

The MNPs-chem and MNPs-leaf have no statistical differences in terms of the inhibition zone produced regardless of the strains tested in these experiments, indicating that the synthesis method did not have an impact on antimicrobial activity (Figure 8a–d). Unlike other authors, in our case, the gold nanoparticles synthesized by any of the methods (chemical or biological) did not show any inhibition in the evaluated strains. It is essential to mention that the leaf extracts did not show bacterial growth inhibition.

## 3. Discussion

In this work, we explore the possibility of using an agro-industrial waste of the *C. chinense* plant to produce AgNPs and AuNPs. We worked exclusively with green synthesis methods, using the aqueous extract of the chilli plant leaf and ultraviolet and microwave radiation sources. Since we used an aqueous extract, the compounds extracted from the leaves were mainly polar, decreasing the families of compounds present in the extract that function as reducing agents or stabilizers of the MNPs. There are works reported in the literature where extracts of fruits and leaves of various plants are used for the formation of MNPs [31,32], in particular in the *C. chinense* plant; most studies are focused on the utilization of the fruit, which is the part that is consumed and is of commercial interest but not in the leaves which are disposed of. We initially evaluated extracts obtained from the stem, root, and leaf of *C. chinense*; however, only leaf extracts were suitable for obtaining MNPs by green synthesis. Although we obtained AuNPs with stem and root extract, nanoparticles were unable to be isolated due to their quick decomposition after synthesis. On the other hand, the extract from the leaves was quite promising, since the polar molecules contained in it (in addition to allowing the formation of nanoparticles) allowed excellent stabilization, acting then as reducing and stabilizing agents.

Although there are works in the literature where there is speculation about the molecules involved in these processes, we decided to study in a little more detail the processes present at the beginning and end of the green synthesis of nanoparticles, with the extracts of the *C. chinense* leaf. We decided to evaluate how the compounds in the extract, with antioxidant power, are affected throughout the synthesis of the nanoparticles. Hypothetically, the antioxidant capacity of the extract should be diminished if those compounds that were originally present in the extract, and that were involved in the reduction of metal ions in the solution, were to give way to the formation of nanoparticles. To do this, the antioxidant capacities were explored at the beginning and end of the reaction, as well as the most important phytochemicals that could be involved in the reduction and stabilization of MNPs.

Various works have described the antioxidant activity of the nanoparticles formed from the green synthesis with phytochemical extracts. In practically all of them, the reduction of the nanoparticles is attributed to the main antioxidant components in the extracts, such as phenolic compounds or flavonoids. However, in different studies, either an antioxidant method is used as a reference (mostly DPPH) [33], or different methods, but with different scales and references [34,35,36,37,38], which makes the interpretation of the data challenging. It is well known that antioxidant activity methodologies can generate complex results to compare due to the kinetics of the methods themselves being diverse or the cut-off point chosen for the measurement depending a lot on the sample [39,40,41]. In this work, we decided to standardize the antioxidant capacity methodologies to measurements where the reaction kinetics had reached a plateau (data not shown), which allowed us to compare the results between the various measurements.

Interestingly, the ABTS and FRAT methods were the most effective for evaluating the antioxidant capacities in our extracts and in the supernatants. It should be noted that both antioxidants have different reaction mechanisms, so the molecules detected by one methodology are not necessarily those detected by the other [42,43]. In the case of the ABTS methodology, it is considered to belong to the so-called SET/HAT family, that is, it shares the Hydrogen atom transfer (HAT) and Single electron transfer (SET) mechanisms, in this type of methodologies, the mechanisms involved depend on the reaction conditions (e.g., pH and type of solvent) and the proportion of the contribution of each mechanism (SET or HAT) varies according to the sample [39,40,41]. On the other hand, the FRAP methodology is considered to work exclusively with the SET mechanism [42,43]. The values obtained in ABTS in this work may be a better reference of how some compounds decrease when being involved in the reduction of nanoparticles because, in a certain way, we are evaluating two mechanisms (SET and HAT) that allow us to detect a greater variety of compounds. On the other hand, unlike other authors [34,35], although we were able to detect activity using the DPPH methodology, it was well below the values with the ones obtained by the other methods of evaluation of antioxidant capacity. Notably, no significant changes were presented at the end of the green synthesis.

In the work of [44], it is proposed that the phenolic compounds and flavonoids present in the *Gnidia glauca* extracts play a role in the synthesis and stabilization of the AuNPs obtained by them, as well as hypothesizing that the polyphenols observed by FTIR could function as stabilizing agents for the nanoparticles generated. We hypothesize that amino acids play a possible role as stabilizing agents in our work. There are reports of the ability of amino acids to selectively coordinate with nanoparticles [45,46,47], which would support our hypothesis. On the other hand, in our experiments, we found that polyphenols and reducing sugars could be involved in synthesizing the AgNPs-leaf and the AuNPs-leaf in our study system, and our data agree with that published by other authors [7,48,49,50,51,52,53,54,55]. Finally, we found an antimicrobial activity of the AgNPs-leaf (obtained by green synthesis) equivalent to the AgNPs-chem (obtained by chemical synthesis) in four bacterial strains (Gram-positive and Gram-negative). The bacteria evaluated in this work have an important implication in human health. All of them are pathogenic in certain conditions for human beings. They are mainly acquired through nosocomial or urinary infections [56,57]. There are reports that several bacterial strains acquire multiple resistances to antibiotics [58], therefore, nanoparticles offer an alternative for their control [38]. However, we did not obtain antibacterial activity with the AuNPs in these same strains, unlike other authors [26,59,60]. These results open the possibility of starting in-depth studies to evaluate the parts typically discarded from *C. chinense* plants to generate added value to this waste or establish synthetic routes that favor the comparative study of the groups of molecules involved in the green synthesis.

## 4. Materials and Methods

### 4.1. Synthesis and Stability of AuNPs-Leaf

#### 4.1.1. Plant Material

Habanero pepper plants (*C. chinense* Jaqc) were used during this work. Seeds were washed with 1%sodium hypochlorite (Industrias Alen, St. Cat, NL, MEX) and rinsed 5 times with distilled water for 5 min each. Later, the seeds were germinated at 26 ± 2 °C in humid chambers. One week after germination, seedlings were individually transplanted into small pots (200 mL) and three weeks later they were transferred to larger pots (1.5 L) in a mixture of peat moss and perlite substrates in a 2:1 ratio, where they were maintained until sample collection. Flowering plants (between 100 and 115 days after germination) were dissected and the samples were separated into three groups: leaf, stem and root. In order to remove excess substrate, roots were submerged and shaken manually in distilled water, three times for 1 min each.

#### 4.1.2. Preparation of *Capsicum chinense* Aqueous Extract

For extracts preparation, 18.04 g, 3.33 g and 24.3 g of fresh chopped stems, roots, and leaves, respectively, were mixed with 30 mL of double-distilled water (ddH_2_O) at continuous stirring on a heat plate at 80 °C for 20 min. After that time, aqueous extracts were filtered through a Whatman general-purpose filter paper by gravity. Later the filtered extract was centrifuged at 4000 rpm for 15 min at room temperature and the solution was filtered through a 0.45 µm nylon Acro disk filter(PALL, Long Island, NY, USA). Filtered extracts from stem and root were diluted to a final volume of 50 mL with ddH_2_O, while the filtered extract from leaves was diluted to a final volume of 250 mL with ddH_2_O. The extracts thus obtained were designated as stem-extract, root-extract and leaf-extract, respectively.

#### 4.1.3. Optimization of AuNPs Synthesis

Using the extracts previously prepared, an experiment was designed where 1 mL of each extract was mixed with three different amounts of HAuCl_4_ (8.05 × 10^−3^, 1.60 × 10^−2^ and 2.4 × 10^−2^ mmol) (Sigma-Aldrich, St. Louis, MO, USA). Afterward, the samples were exposed to UV light radiation during: 5, 30, 60, 90, 120, 180, 210, 240, 270 and 300 min. After each exposure time, samples were analyzed by UV-vis spectroscopy.

A second experiment was performed using 1 mL of extract and the same amounts of HAuCl_4_ tested above (8.05 × 10^−3^, 1.60 × 10^−2^ and 2.4 × 10^−2^ mmol), but this time, the samples were exposed to microwave radiation during 15 s. Samples were analyzed by UV-vis spectroscopy as soon as reaction time was completed and one hour after synthesis.

A third experiment was conducted in order to optimize the yield of AuNPs synthesis. Only the leaf extract was used under microwave radiation, due to previous experimental results. Three different volumes of leaf extract (1 mL, 3 mL and 6 mL) were mixed with 8.05 × 10^−3^ mmol of HAuCl_4_. Subsequently, the samples were exposed to microwave radiation for 15 s.

All UV-vis spectra were obtained using a Thermo Scientific™ Multiskan SkyHigh Microplate Spectrophotometer (Thermo Fisher Scientific, Waltham, MA, USA) at a wavelength range of 250 to 1000 nm.

#### 4.1.4. Stability of AuNPs-Leaf

Once the synthesis parameters of AuNPs-leaf were established, the nanoparticles were centrifuged and washed with ddH_2_O three times for their purification. Subsequently, the solution with the AuNPs-leaf was monitored for stability. For this purpose, the same sample was analyzed by UV-vis absorption spectroscopy. In addition, two samples of the AuNPs-leaf were collected: one on the same day of their synthesis and the other 15 days after synthesis and transmission electron microscopy (TEM) was performed.

UV-vis absorption spectra were carried out at room temperature using a Thermo Scientific™ Multiskan SkyHigh Microplate Spectrophotometer at a wavelength range of 250 to 1000 nm for AuNPs-leaf.

Transmission electron microscopy (TEM) analysis was performed using a JEOL JEM-1010 (JEOL, Ltd., Tokyo, Japan) unit operated at 90 kV. The preparation of the samples consisted of depositing a drop of it on a 400-mesh copper grid, which was left at room temperature for one day to remove moisture.

### 4.2. Synthesis and Stability of AgNPs-Leaf

#### 4.2.1. Optimization of AgNPs-Leaf Synthesis

The same leaf extract described above was used for the synthesis of AgNPs-leaf. Two sources of radiation were evaluated again, microwave radiation and UV radiation. For both syntheses, 1 mL of leaf extract was mixed with 8.05 × 10^−3^ mmol of AgNO_3_ (Sigma-Aldrich, St. Louis, MO, USA).

The exposition times tested were: 5, 10, 15 and 20 s for microwave radiation and 5, 10, 15, 20, 30 and 60 min for UV radiation.

#### 4.2.2. Stability of AgNPs-Leaf

Once the synthesis parameters of AgNPs-leaf were established, the nanoparticles were centrifuged and washed with ddH_2_O three times for their purification. Subsequently, the solution with the purified AgNPs-leaf was monitored for stability. For this purpose, the same sample was analyzed by UV-vis absorption spectroscopy. In addition, two samples of the AgNPs-leaf were collected: one on the same day of their synthesis and the other 15 days after synthesis and transmission electron microscopy (TEM) was performed.

The morphology and size of the AgNPs-leaf were obtained using a TEM JEOL JEM 1010 equipment operated at 90 kV. UV-vis absorption spectra were carried out at room temperature in a Thermo Scientific™ Multiskan SkyHigh Microplate Spectrophotometer at a wavelength range of 250 to 1000 nm for AgNPs-leaf.

### 4.3. Characterization by SEM-EDX

The characterization of the nanoparticles by ultra-high-resolution scanning electron microscopy (UHR-SEM), was carried out using a UHR-SEM, Dual Beam Helios Nanolab 600 (FEI, Hillsboro, OR, USA) coupled to an EDX detector. The analysis was carried out with a voltage of 15 kV. The preparation of the samples was carried out by putting a drop of the sample on double-sided conductive carbon tape, placed in an aluminum sample holder.

### 4.4. Antioxidant Activities of Leaf Extract and Nanoparticles Supernatants

#### 4.4.1. Supernatants Obtention

After synthesis of AuNPs-leaf and AgNPs-leaf, they were centrifuged at 13,000 rpm for 15 min. Subsequently, the supernatants were separated from the nanoparticle pellets. Supernatants thus obtained were stored at 4 °C for further analysis.

#### 4.4.2. ABTS Antioxidant Assay

The ABTS (2,2′-azino-bis-3-ethylbenzothiazoline-6-sulfonic acid) (Sigma-Aldrich, St. Louis, MO, USA) cation radical scavenging activity of supernatants and the extracts were measured. The ABTS assay was conducted as described by [61,62] with slight differences. In brief: 7 mM ABTS solution was mixed with 245 mM potassium persulphate (Sigma-Aldrich, St. Louis, MO, USA) prepared using ddH_2_O (working solution). The mixture was incubated for 16 h. After incubation, the solution was diluted until the absorbance at 754 nm becomes 0.7. A standard curve was prepared with Trolox (6-hydroxy-2,5,7,8-tetramethylchroman-2-carboxylic acid) (Sigma-Aldrich, St. Louis, MO, USA). The reaction was carried out in a microplate, mixing 20 µL of the sample with 280 µL of ABTS working solution. Measurements were performed in triplicate at 754 nm for 30 min.

#### 4.4.3. DPPH Radical Scavenging Activity

The DPPH (2,2-diphenyl-1-picrylhydrazyl hydroxyl radical) (Sigma-Aldrich, St. Louis, MO, USA) assay was performed according to [63], protocol. In brief, a 1.6 mM DPPH solution was prepared (working solution). The calibration curve was performed using Trolox as a reference. The reaction was carried out in a microplate, mixing 20 µL of the sample with 280 µL of working solution. Measurements were performed in triplicate at 515 nm for 30 min.

#### 4.4.4. Ferric Reducing Antioxidant Power Assay

FRAP (Ferric reducing antioxidant power assay) assay was performed following the protocol described by [64]. Briefly, the working solution was prepared by mixing: 2.5 mL of a 10 mmol/L TPTZ (2,4,6- tripyridyl-s-triazine) (Sigma-Aldrich, St. Louis, MO, USA) solution in 40 mmol/L HCl (Karal, Leon, Gto, MEX) plus 2.5 mL of 20 mmol/L FeCl3 (Sigma-Aldrich, St. Louis, MO, USA) and 25 mL of 0.3 mol/L (pH 3.6) acetate buffer, and was prepared freshly and warmed at 37 °C. The analysis was performed by mixing 50 µL of sample and 100 µL of the working solution by triplicate. The reaction was monitored at 595 nm in the spectrophotometer after 30 min.

As a standard for the three antioxidant tests, a series of Trolox dilutions in a range of 0–400 μM was used as a standard curve. The results were expressed as μmol Trolox equivalent. A Thermo Scientific™ Multiskan SkyHigh Microplate Spectrophotometer was used as a reader.

### 4.5. Phytochemical Screening

#### 4.5.1. Total Reducing Sugars

The determination was carried out under the 3,5-dinitrosalicylic acid (DNS) (Sigma-Aldrich, St. Louis, MO, USA) reduction test by the Miller method [65,66]. For this, 0.5 mL of sample were mixed with 0.5 mL of the working reagent (1% DNS). This mixture was boiled at 99 °C for 5 min, then 4 mL of ddH_2_O were added. Each sample was monitored at 575 nm. As standard, glucose (Sigma-Aldrich, St. Louis, MO, USA) was used in a range between 0 and 1 g/L. Results were expressed as mM of Glucose equivalent (GluE). Experiments were performed in triplicate.

#### 4.5.2. Total Flavonoids

The quantification of total flavonoids was carried out by the aluminum chloride (AlCl_3_) (Golden Bell, Zap, JAL, MEX) method [67]. A quercetin (Sigma-Aldrich, St. Louis, MO, USA) standard curve of 0–1000 µg/mL was prepared. An aliquot of 1 mL of sample or standard was mixed with 0.2 mL of 10% (*w*/*v*) AlCl3 solution in methanol (Hycel, Zap, JAL, MEX), 0.2 mL (1 M) of potassium acetate (Sigma-Aldrich, St. Louis, MO, USA) and 5.6 of mL distilled water. The mixture was incubated for 30 min at room temperature followed by the measurement of absorbance at 510 nm. The outcome data were expressed as mM EQ (millimolar equivalent of Quercetin). Experiments were performed in triplicate.

#### 4.5.3. Total Polyphenols

The determination was made based on the Folin–Ciocalteu method [68]. A total of 125 µL of each sample were mixed with 750 µL of double distilled water and 62.5 µL of Folin–Ciocalteu reagent (Golden Bell, Zap, JAL, MEX). This mixture was incubated in the dark for 5 min, and immediately 250 µL of ddH_2_O and 187.5 µL of 20% Na_2_CO_3_ (Merck, Kenilworth, NJ, USA) were added. Solutions were gently mixed and incubated for two hours in the dark at room temperature. Afterward, samples were measured at 765 nm. The outcome data were expressed as mM GAE (millimolar of gallic acid equivalent) (Sigma-Aldrich, St. Louis, MO, USA). Experiments were performed in triplicate.

#### 4.5.4. Total Proteins

Total protein content was determined by the Bradford method [69]. Briefly: 200 µL of Bradford Reagent (Biorad^®^, Hercules, CA, USA) was placed in a 96-microplate well, and 5 µL of each sample or standard was added. After 10 min of incubation at room temperature. The microplate was monitored at 595 nm. A standard curve made of bovine serum albumin (BSA) (Sigma-Aldrich, St. Louis, MO, USA) was used. Experiments were performed in triplicate.

#### 4.5.5. Free Amino Acids

Free amino acids were quantified by Ninhydrin method [70]. A standard curve from 0 to 250 µM of glycine was used. Analysis was performed by mixing 200 µL of sample or standard and 100 µL of ninhydrin (Sigma-Aldrich, St. Louis, MO, USA). The mixture was boiled for 10 min and immediately the reaction was stopped by the addition of 500 µL of 95% ethanol (Hycel, Zap, JAL, MEX). Each sample was read at 570 nm. Experiments were performed in triplicate.

### 4.6. Antimicrobial Activities

The antimicrobial tests were carried out by triplicate based on the diffusion in plaque technique, according to [62]. In brief: an inoculum was prepared at an optical density of 0.6 ± 0.05 at 600 nm in Tryptic Soy Broth (TSB) (MCD LAB, Tlalnepantla, EDO MEX, MEX) of the following bacteria: Gram-positive: *Staphylococcus aureus*, *Enterococcus faecalis* and Gram-negative: *Escherichia coli* and *Serratia marcescens*. Subsequently, 175 µL of the inoculum were added to 25 mL of molten Tryptic Soy Agar (BD Bioxon, CdMX, MEX) and poured into sterile Petri dishes. Once the agar solidified, wells of approximately 6 mm in diameter were made. The boxes were initially incubated at 16 °C for 24 h to allow diffusion. Subsequently, they were incubated at 37 °C for 24 h. A total of 20 µL of Kanamycin (35 µg/mL) (GoldBio, Olivette, MO, USA) were placed in the center of each box as a positive control and 20 µL of 4.0 mM of NPs-chem and NPs-leaf were used.

### 4.7. Characterization by FTIR-ATR and XPS of AgNPs-Leaf and AuNPs-Leaf

X-ray Photoelectron Spectroscopy (XPS) analysis, were made on an XR50 M monochromatic Al Kα1 (hν = 1486.7 eV) X-ray source and a Phoibos 150 spectrometer, this equipment has a one-dimensional detector 1D-DLD, provided by SPECS (Berlin, Germany). The samples were mounted on steel sample holders using double-sided copper tape. They were previously precipitated and left to dry for two days, for the total elimination of humidity. Measurements were made at 250 Wy 12.5 kV and at an operating pressure of 2.29x.

FTIR-ATR were performed using a Thermo Scientific, Nicolet iS5 Id5 ATR infrared spectrometer, equipped with a diamond-ATR-accessory. The spectra were acquired in transmittance/absorbance mode at 4 cm^−1^ resolution and 20 scans.

### 4.8. Statistical Analysis

All values were obtained from three independent analyses. The results expressed as mean ± standard deviation. A one-way analysis of variance (ANOVA) test was used for statistical data analysis. Computations of Tukey post-hoc means comparisons were included to evaluate the statistical significance of the recorded differences among means. Data within the same column or row for tables or bars (in the case of charts), sharing the same superscripts or letters, are not significantly different at *p* < 0.05. Data within the same column, row or bars, sharing different superscripts or letters are significantly different at *p* < 0.05. The statistical data processing was performed using software Statgraphics 19 ^®^ (Statgraphics Technologies, Inc., The Plains, VA, USA).

## Figures and Tables

**Table 1:** Inhibition zone growth (mm).

Strain	Sample	Inhibition Zone Diameter (mm)
*S. marcescens*	AgNPs-chem	9.57 ± 0.39 ^a^
	AgNPs-leaf	9.96 ± 0.43 ^a^
*E. faecalis*	AgNPs-chem	5.85 ± 0.22 ^b^
	AgNPs-leaf	5.38 ± 0.26 ^b^
*E. coli*	AgNPs-chem	8.49 ± 0.17 ^c^
	AgNPs-leaf	8.64 ± 0.12 ^c^
*S. aureus*	AgNPs-chem	9.82 ± 0.43 ^a^
	AgNPs-leaf	10.22 ± 0.46 ^a^

Values are expressed as mean ± standard deviation (*n* = 3) Positive control with kanamycin was used as a standard antibiotic, showing an inhibition zone of 21.39 ± 3.69 mm. Values with different superscript letters (^a^, ^b^, ^c^) in each column are significantly different at *p* < 0.05.

## Data Availability

Not applicable.

## Supplementary Materials

The following are available online, Figure S1: UV-vis absorption spectra of AuNPs at different times of microwave light radiation. Figure S2: UV-vis absorption spectra of AgNPs-leaf at different times of UV light radiation.

## Sample Availability

Samples of the compounds are available from the authors.
