# Supplementary material for: Green Synthesis of Gold and Silver Nanoparticles Using Leaf Extract of Capsicum chinense Plant
Source: Molecules. 2022 Mar 4;27(5):1692. doi: 10.3390/molecules27051692 (PMC8911899; doi:10.3390/molecules27051692)
Supplement: Supplementary file 1 [file molecules-27-01692-s001.zip › molecules-1574713-supplementary.pdf]

Green synthesis of gold and silver nanoparticles with leaf extract of Capsicum chinense plant

Diego Alberto Lomelí-Rosales[1], Adalberto Zamudio-Ojeda [2], Oscar Kevin Reyes-Maldonado [1], Morelia Eunice Ló-pez-Reyes [1], Georgina Cristina Basulto-Padilla [1], Edgar José Lopez-Naranjo [3], Víctor Manuel Zuñiga-Mayo [4*] and Gilberto Velázquez-Juárez [1*]

1 Departamento de Química, Centro Universitario de Ciencias Exactas e Ingenierías, Universidad de Guadala-jara. Blvd. Marcelino García Barragán #1421, C.P, 44430, Guadalajara, Jalisco, México; diego.lomeli4077@aca-demicos.udg.mx (D.A.L-R.); oscar.reyes@alumnos.udg.mx (O. K: R-M.); morelia.lopez@academi-cos.udg.mx (M. E. L-R); georgina.basulto@alumnos.udg.mx (G. C B-P.); gilberto.velazquez@academi-cos.udg.mx (G. V-J.).

2 Departamento de Física, Centro Universitario de Ciencias Exactas e Ingenierías, Universidad de Guadala-jara. Blvd. Marcelino García Barragán #1421, C.P, 44430, Guadalajara, Jalisco, México; adalberto.zojeda@aca-demicos.udg.mx (A. Z-O.)

3 Departamento de Ingeniería de Proyectos, Universidad de Guadalajara, José Guadalupe Zuno # 48, Zapopan, Jalisco, 45101, México; edgar.lopezn@academicos.udg.mx (E. J. L-N.).

4 CONACyT-Instituto de Fitosanidad, Colegio de Postgraduados, Campus Montecillo, Texcoco, Estado de Mé-xico, México. zuniga.victor@colpos.mx (V. M. Z-M.). * Correspondence: gilberto.velazquez@academicos.udg.mx (G. V-J.); zuniga.victor@colpos.mx (V. M. Z-M.).

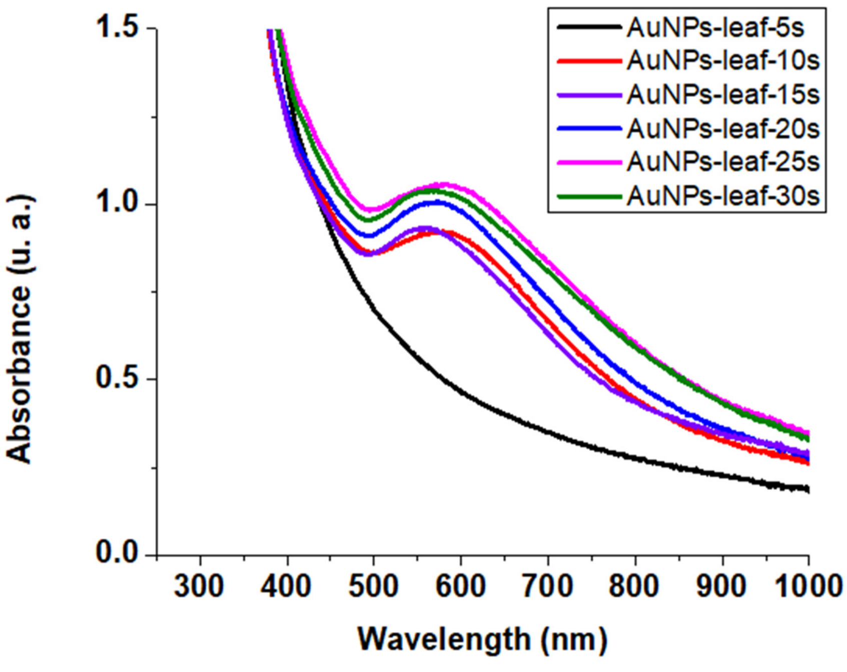

Figure S1. UV-vis absorption spectra of AuNPs at different times of microwave light radiation.

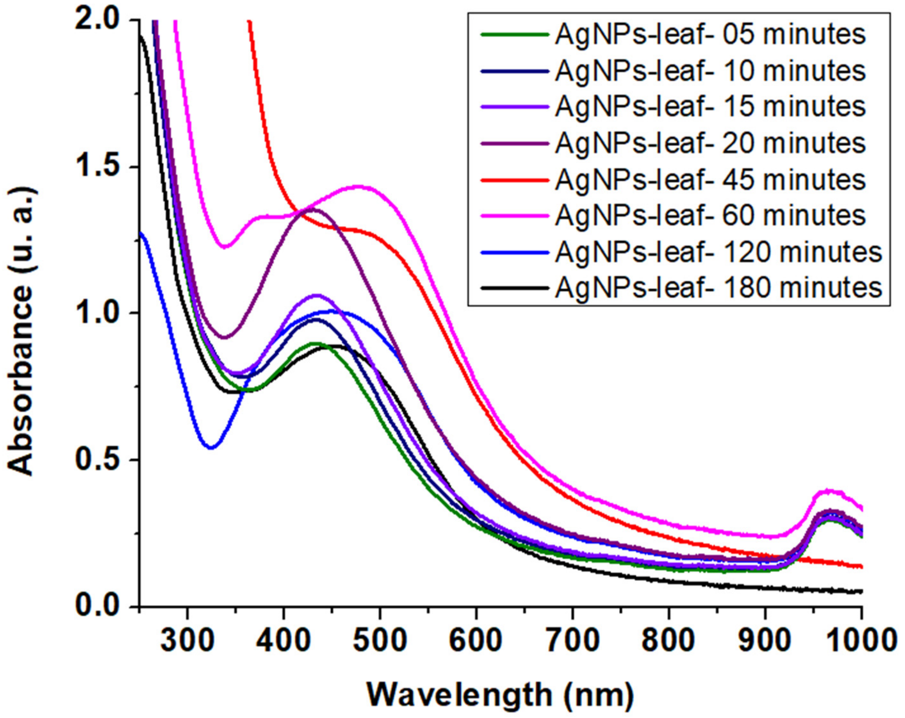

Figure S2. UV-vis absorption spectra of AgNPs-leaf at different times of UV light radiation.
